# Factors affecting left ventricular remodeling after valve replacement for aortic stenosis. An overview

**DOI:** 10.1186/1476-7120-4-25

**Published:** 2006-06-27

**Authors:** Emmanuel Villa, Giovanni Troise, Marco Cirillo, Federico Brunelli, Margherita Dalla Tomba, Zen Mhagna, Giordano Tasca, Eugenio Quaini

**Affiliations:** 1 Cardiac Surgery Unit, Cardiovascular Dept. *Poliambulanza Foundation Hospital, *Brescia, Italy; 2 University of Milan, Milan, Italy

## Abstract

Although a small percentage of patients with critical aortic stenosis do not develop left ventricle hypertrophy, increased ventricular mass is widely observed in conditions of increased afterload. There is growing epidemiological evidence that hypertrophy is associated with excess cardiac mortality and morbidity not only in patients with arterial hypertension, but also in those undergoing aortic valve replacement. Valve replacement surgery relieves the aortic obstruction and prolongs the life of many patients, but favorable or adverse left ventricular remodeling is affected by a large number of factors whose specific roles are still a subject of debate. Age, gender, hemodynamic factors, prosthetic valve types, myocyte alterations, interstitial structures, blood pressure control and ethnicity can all influence the process of left ventricle mass regression, and myocardial metabolism and coronary artery circulation are also involved in the changes occurring after aortic valve replacement. The aim of this overview is to analyze these factors in the light of our experience, elucidate the important question of prosthesis-patient mismatch by considering the method of effective orifice area, and discuss surgical timings and techniques that can improve the management of patients with aortic valve stenosis and maximize the probability of mass regression.

## Review

Left ventricular pressure overload due to aortic valve stenosis (AS) leads to a marked hypertrophic response of the myocardium, which is probably an adaptative reaction aimed at normalizing the increased wall stress. Although a small percentage of patients with critical AS do not develop left ventricle (LV) hypertrophy, increased LV thickness is widely observed in conditions of increased afterload and is usually accompanied by a parallel deposition of new sarcomers. This compensatory response seems to maintain cardiac performance despite the high intracavitary systolic pressure [1,2].

AS is a common disorder and the most frequent acquired valvular disease in developed countries. The natural history of symptomatic patients is dismal, and even asymptomatic subjects with a significant stenosis face a risk of sudden death that has been reported to be ~1% per year [3,4]. Hypertrophy is common to pressure overload conditions such as arterial hypertension, AS, aortic coartaction and hypertrophic obstructive cardiomyopathy, and there is increasing epidemiological evidence that it is associated with excess cardiac mortality and morbidity [5-9]. Moreover, in isolated AS, it has recently been shown that increased LV mass alone predicts systolic dysfunction and heart failure regardless of the severity of the valvular obstruction. For this reason, LV hypertrophy can be interpreted as being a synonymous with a maladaptive response to aortic valve disease rather than a compensatory reaction [10]. Aortic valve replacement (AVR) surgery dramatically changes the clinical course of patients with AS by relieving the high pressure gradient and allowing the reversal of the LV hypertrophic process. Age-corrected survival has been reported to be nearly normal after AVR [11], but there are still some questions as to whether the ventricular chamber can return to its normal size, and how rapidly myocardial hypertrophy and LV dysfunction regress.

What follows is an experience-based review of the factors involved, and the extent to which the myocardium itself may recover (*favorable remodeling*) or deteriorate (*adverse remodeling*).

## Age

The prevalence of calcific AS increases with age (2–4% of adults aged more than 65 years) and, as the majority of patients suffering increased mortality and morbidity due to aortic valve disorders are elderly [12], it is extremely important to know whether they may benefit from AVR and if favorable LV remodeling is probable [13]. Hanayama *et al*. have recently reported that age is not a determinant of incomplete mass regression after a mean follow-up of 3.75 years [14], and Gaudino *et al*. have published a similar finding [15]. Using more accurate 3-D echocardiography, Kühl *et al*. consistently observed that normalization of the LV mass index after one year was not related to age at the time of surgery [16], but the results of studies by Lund *et al*., who developed a preoperative prognostic index specifically conceived for patients with AS undergoing AVR that included age, indirectly suggest that age is associated with the LV mass index after 10 years: the higher scores correlated with a higher LV mass index during the postoperative course [17]. Univariate and multivariate analysis of our own patient series have not indicated age as a factor influencing the process of mass regression even in the subgroup of patients with a prosthesis-patient mismatch (PPM) [18,19].

## Gender

It is known that there is a gender-related difference in the development of pressure overload-induced LV hypertrophy: after adjusting for body surface area, females have less mass, more concentric hypertrophy, less wall tension, fewer alterations in passive elastic properties, higher ejection fractions and smaller LV volumes [20-22]. The effect of these gender-related differences in hypertrophy patterns on the recovery and regression of the LV mass index is still being debated. In their medium-term study, Hanayma *et al*. found that the LV hypertrophy index of females was less likely to regress incompletely [14], and we have found that female gender is an independent predictor of greater LV mass regression except in the particular subgroup of patients with PPM, in whom it plays no predictive role [18,19]. In the prognostic index developed by Lund *et al*., female gender is a neutral factor whereas male gender adds one point: i.e. it increases the risk of post-AVR mortality and morbidity [17]. On the contrary, Del Rizzo *et al*. found that male gender was an independent predictor of LV mass regression after AVR with stentless bioprostheses [23] although, some years later, Gelsomino *et al*, using another type of stentless xenograft, found that it negatively affected LV mass regression [24]. However, gender differences in LV adaptation do not seem to influence survival after AVR [15,21,25,26]. Finally, the results of the 3-D echocardiography study by Kuhl *et al*. indicate that 1-year LV mass index normalization is unrelated to gender [16].

## Hemodynamic factors

The hemodynamic advantage of AVR arises from its ability to minimize postoperative gradients and favor the normalization of LV mass and function but, although it intuitively seems to be quite important, the influence of hemodynamic variables on the extent of LV mass regression is controversial.

A PPM is considered such when the effective orifice area (EOA) of the implanted prosthesis is less than that of the normal human valve: i.e. too small in relation to body surface area (BSA). This is a crucial parameter when evaluating the performance of valve substitutes: some authors have found that PPM leads to higher mortality rates [27,28] and others have found no effect on overall survival [14,15,29], but there is considerable evidence that it has detrimental implications in terms of LV workload [19,23,29,30]. Moreover, its clinical impact seems to be related to both its severity and LV function, thus underlining the fact that a diseased ventricle is much more sensitive to increased afterload [27]. Indexed EOA (EOA divided by BSA) is decidedly a more physiological parameter to adopt in defining PPM, whereas the labeled or internal geometric size of the prosthesis may be misleading [31,32]. It has been demonstrated that, in order to avoid any significant gradient at rest or during exercise, the indexed EOA of a prosthetic aortic valve should ideally be no less than ~0.8–0.9 cm^2^/m^2^. However, in clinical practice, post-AVR indexed EOA be less than this for a number of reasons: the size of the aortic annulus may be reduced because of calcifications, fibrosis, hypertrophy in the LV outflow tract, or because the structural support of the valve prosthesis may be quite bulky (especially in older models) and create a relative obstruction to flow. Moreover, the procedures for implanting an adequate prosthesis in a small and severely calcified aortic root (annulus enlargement, root replacement, LV outflow enlargement) can be technically more difficult and often require a longer period of aortic cross-clamping. They may also be contraindicated in some situations: for example, the presence of heavy and extended calcifications around the coronary ostia does not allow root replacement and coronary artery reimplantation. Consequently, the perceived balance between the increased preoperative risk of the more complex operation needed to avoid a potentially suboptimal late clinical outcome due to a small prosthesis, and the chances of the patient experiencing meaningful long-term survival and quality of life determines the surgeon's choice of prosthesis type and size.

**Figure 1:**
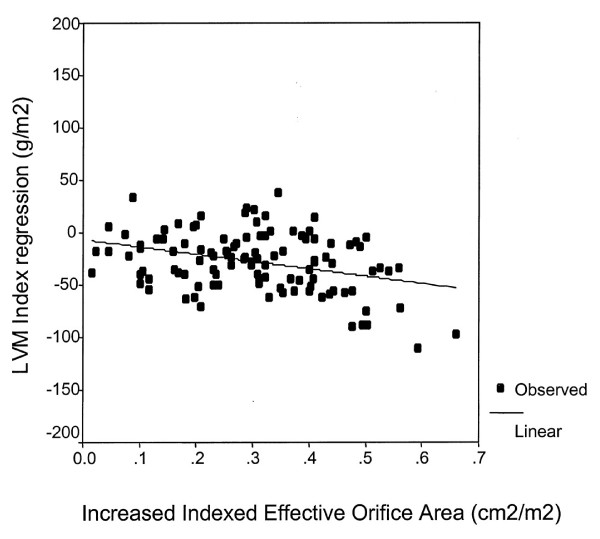
**Influence of the relative increase of EOA after AVR on LV mass**. Correlation between absolute left ventricular mass (LVM) index regression and increased indexed effective orefice area (r = -0.31; r^2 ^= 10%, p = 0.001). *(Reprinted from Annals of Thoracic Surgery, Vol. 79, Tasca G et al, Impact of the improvement of valve area achieved with aortic valve replacement on the regression of left ventricular hypertrophy in patients with pure aortic stenosis, Page 1294, © 2005, with permission from The Society of Thoracic Surgery) *[19].

The true incidence of PPM can only be discovered using the EOA method to evaluate the prosthesis performance. This has repeatedly shown that the presence of PPM seriously hampers LV mass regression [18,23], whereas older comparisons based on labeled prosthesis size found prostheses of different sizes led to similar degrees of LV mass reduction even in the case of PPM [33,34]. One expression of the potential severity of a mismatch is given by the relationship showing that the transvalvular gradient increases exponentially with a decrease in indexed EOA. We have found an independent relationship between indexed EOA and the extent of LV mass regression after AVR. Furthermore, the pattern of LV remodeling was influenced by PPM, with a smaller decrease in chamber internal dimension in patients with than in those without a mismatch [18]. We also found that the degree of mass regression may vary markedly from one patient to another. Some patients with PPM may therefore show a substantial regression in LV hypertrophy despite the presence of a relatively high residual transvalvular gradient because the regression in LV mass in such patients is independently influenced by the relative increase in valve EOA achieved by AVR (Fig. 1) [19]. This explains why some authors have reported that patients with PPM and/or small prostheses show significant reductions in LV mass.

In conclusion, even in the presence of PPM, surgery usually improves hemodynamics. The extent of the improvement can be quite important and it is likely that the best results can be expected if PPM is completely avoided. Moreover, the relationship between gradients and indexed EAO is curvilinear, and the implications for a given patient are directly related to his/her original and final positions on the indexed EAO-gradient curve [30,31]. One practical approach to reduce the impact of PPM is to begin by determining preoperatively the minimum EOA that the implanted prosthesis should have to avoid moderate-to-severe PPM. This is done by multiplying the patient's BSA (calculated on the basis of his/her weight and height) by 0.85 cm^2^/m^2^: for example, if the BSA is 1.7 cm^2^/m^2^, the minimum EOA is 1.7*0.85 = 1.44 cm^2^. The next step is verifying which of the available prostheses have the same or a larger EOA by looking at the widely available published data reported in the literature or provided by the manufacturers (Tab.1) [27,30]. In this way, at the time of the operation, the surgeon can attempt to implant one of the selected prostheses or, if technically possible, perform an aortic root enlargement or other procedure to enable the accommodation of a valve with the adequate EOA.

## Valve type

Pibarot *et al*. have reported the relative positions of different types of biological aortic valve substitutes on the exponential curve linking the transvalvular gradient and indexed EOA [30]. The majority of patients with a stented prosthesis have an indexed EAO of ≤ 0.85 cm^2^/m^2 ^and are therefore on the steep portion of the curve, where the gradients are relatively high, whereas most patients with a stentless prosthesis and almost all of those undergoing an aortic homograft or pulmonary autograft have a larger indexed EOA and are on the flat portion of the curve, where the gradients are relatively low [30,35]. It is possible that the consequences on LV mass regression may eventually be predicted on the basis of these findings, but it is currently hazardous to do so for a number of reasons. Firstly, there is a lack of randomized prospective studies of homogeneous cohorts relating valve types and their benefits in terms of mass reduction. Secondly, there are differences in the indications, availability and technical risks related to the various valve substitutes: i.e. the hypothetical superiority of mechanical prostheses in terms of hypertrophy regression does not change the indication for a biological prosthesis in the elderly. Thirdly, recently published studies have failed to demonstrate any robust advantage of a particular type of substitute. Like Gaudino *et al*. very recently [15], Hanayma *et al*. found that the type of prosthesis did not predict the extent of postoperative LV mass regression in a long-term prospective study comparing stented porcine valves, stented pericardial prostheses, stentless porcine valves, and tilting and bileaflet mechanical prostheses [14]; furthermore, Kühl *et al*. found that 1-year normalization of the LV mass index studied by means of 3-D echocardiography was not related to valve type [16], and a tentative meta-analysis of 501 patients by Sharma *et al*. revealed no substantial advantage of stentless over stented valves in terms of the rate of LV mass regression [26]. Also in prospective, multicenter randomized comparisons there were similar reductions in LV mass at 12 months with both stented and stentless valves despite significant differences in indexed EOA and peak flow velocity in favor of the stentless valves [Circ 2005]. We have studied stented and stentless biological valves and mechanical prostheses in the challenging subgroup of patients with PPM and concluded that valve type was not one of the factors influencing mass regression [19]. In fact, we feel that other factors must also be considered (see Non-hemodynamic factors and Conclusions) and that any comparisons must always be made at homogeneous values of indexed EOA because stratification by prosthesis diameter or size is probably erroneous.

## Myocardial metabolism

It has been shown that LV hypertrophy can be accompanied by alterations in myocardial high-energy phosphate metabolism [36], but it is only recently that the availability of magnetic resonance (MR) spectroscopy has made it possible to study these alterations after AVR [37]. Changes in myocardial high-energy phosphate metabolism are usually expressed as changes in the phosphocreatine-to-adenosine triphosphate (PCr-ATP) ratio, which is reduced in AS pressure overload. Beyerbacht *et al*. attributed pre-AVR findings of a reduced ratio at rest to myocardial stress and ischemia: i.e. a hypertrophy-induced imbalance between myocardial oxygen supply and demand. Consequently, a recovery in the post-AVR myocardial PCr-ATP ratio accompanied by a reduction in the LV mass index (as revealed by studying LV geometry and function) may indicate that the reduced pressure overload has decreased the metabolic demand of the myocardium and improved coronary blood flow. The same authors also reported a statistically significant correlation between myocardial high-energy phosphate metabolism and LV diastolic function [37].

## Diastole

Doppler echocardiographic alterations in LV diastolic function occur early under conditions of pressure overload and precede the increase in LV mass. It is not clear whether the early reduction in afterload immediately after AVR (when hypertrophy is still present) also leads to improved diastolic function. Assessments of the time constant of relaxation, peak filling rate and the constant of myocardial stiffness by Villari *et al*. after 89 ± 21 months have shown that diastolic function normalizes only late after AVR [38]. This indicates that the process of favorable remodeling (i.e. the regression of myocardial hypertrophy *and *interstitial fibrosis) is slow and may allow diastolic normalization only after its completion. Hess *et al*. previously made another important contribution based on endomyocardial biopsies obtained before and after surgery, and simultaneous echocardiography and pressure measurements, which showed that diastolic alterations persist after AVR due to increased myocardial stiffness [39]. Their findings of a decrease in muscle fiber diameter and a relative increase in interstitial fibrosis, without any change in fibrous content, showed that the post-AVR regression of myocardial hypertrophy was accompanied by an increase in myocardial stiffness due to the relatively slower remodeling of the extracellular compartment. These conditions are not incompatible with the full normalization of the diastolic parameters described by Villari *et al*. because the follow-up of their study was longer.

Another contribution comes from Ikonomidis *et al*., who assessed the effect of residual pressure overload on the regression of LV hypertrophy and its relationship to diastolic function two months and four years after AVR [40]. Isovolumic relaxation significantly decreased from 93 ± 20 ms to 78 ± 12 ms to 81 ± 15 ms, and deceleration time from 241 ± 102 ms to 205 ± 77 ms to 226 ± 96 ms. The prolonged isovolumic relaxation time was associated with significant septal and posterior wall hypertrophy, whereas the prolonged deceleration time was related to a high residual gradient. They concluded that LV diastolic function improves early after surgery in parallel with the reduction in the aortic gradient, but prolonged Doppler indices of myocardial relaxation and ventricular filling were observed in patients with significant LV hypertrophy and a residual pressure gradient soon after surgery. They also reported that diastolic function remained improved four years postoperatively [40].

In a recent longer follow-up study of a large and representative population of patients with a mean age of 67 ± 8.6 years, Gjertsson *et al*. [41] evaluated diastolic function by integrating mitral and pulmonary venous flow data. The patients were divided into two groups on the basis of whether their filling pattern indicated normal/mild or moderate/severe diastolic dysfunction. Eighty-three percent of the patients showed signs of LV hypertrophy preoperatively; this had decreased to 29% (p < 0.001) after two years but no further decrease was found after 10 years. Deceleration time decreased during the follow-up, whereas the E/A and S/D ratios increased. The percentage of patients with moderate/severe diastolic dysfunction remained unchanged between the preoperative and 2-year examinations (7% *vs *13%; p = 0.27), but increased after 10 years (61%; p < 0.0001). Although the findings regarding the degree of LV mass reduction agree with those of other investigators [38,40], the prevalence of disturbed diastolic function was unexpected and related by the authors to the older age of their study cohort. This may also imply a more advanced degree of interstitial fibrosis due to longer exposure to pressure overload, and a consequently limited possibility of favorable remodeling: i.e. severe diastolic dysfunction indicates non-reversible myocardial changes. Although distinguishing the effects of age and long-term exposure to increased afterload is important in terms of AVR timing, it has still not been done. Gjertsson *et al*. did not make a specific analysis in relation to PPM (see Hemodynamic factors), but the patients with the worst diastolic function after 10 years had a significantly higher prosthesis gradient [41]. This indirectly further underlines the importance of avoiding PPM in order to optimize outcomes.

## Ejection fraction

The effect of LV systolic function on mass regression has rarely been investigated in detail. Lund *et al*. performed transmural biopsies during AVR and found that the preoperative LV ejection fraction (EF) inversely correlated with myocyte nucleus volume and the fibrous tissue, muscle cell and LV mass indices. The LV mass index 18 months after AVR was significantly related to the above mentioned morphological parameters, thus suggesting that favorable remodeling after the removal of the hypertrophy trigger may be predetermined by profound changes in hypertrophied myocytes and ventricular fibrosis in many patients [42]. Kühl *et al*. consistently found that LV mass index studied by means of 3-D echocardiography was less likely to normalize one year after AVR in patients with a reduced preoperative EF [16]. Our own early follow-up findings do not indicate EF as a factor influencing LV mass regression, and Hanayama *et al*. also failed to find any difference in preoperative EF between the patients with a normal or abnormal LV mass index after a longer follow-up [14,19]. However, particular attention is required in the subgroup of patients with severe ventricular dysfunction and certain surgical strategies may promote LV recovery [35].

## Coronary circulation

Epicardial coronary arteries are larger in patients with aortic valve disease, but it has been reported that the appropriateness of their cross-sectional areas normalized on the basis of muscle mass is inadequate, and this contributes to explain the anginal symptoms that occur in AS: after AVR, reduced LV hypertrophy and smaller coronary arteries allow a more adequate match of coronary size and muscle mass [43]. However, in addition to alterations in epicardial arteries, abnormalities in microcirculatory function may play a major role in causing the reduced coronary vasodilator reserve and subendocardial ischemia typically observed in AS. Reduced diastolic perfusion, and increased systolic impedance to coronary flow due to perivascular compression, are considered to be primary contributors to impaired coronary microcirculatory function, mainly because of the reduction in maximal myocardial blood flow. The role of favorable post-AVR LV remodeling in the coronary microcirculation has recently been investigated by means of positron emission tomography and MR. The conclusion was that changes in microcirculatory function did not directly depend on LV mass regression, and it was suggested that reduced extravascular compression and an increased diastolic perfusion time may be the main mechanisms improving hyperemic myocardial blood flow and restoring coronary vasodilatation reserve after AVR [44]. Cheaper, reliable and more accessible non-invasive tools than positron emission tomography are now gaining acceptance as a means of exploring coronary microcirculation impairment, and it should not be long before further documentation of post-surgical vascular remodeling is available [45]. AS shares many risk factors with atherosclerotic coronary artery disease (CAD), and it is known that concomitant coronary artery bypass grafting increases the operative risk of AVR. Biederman *et al*. have recently reported that CAD also has a negative impact on reverse remodeling, as revealed by means of the very promising method of intramyocardial MR imaging [46]. They inferred that the presence of CAD is sufficient to delay LV mass regression via a number of putative pathways, notably an inability to reset mRNA signaling and a failure to inactivate the metalloproteinases that promote interstitial fibrosis and blunt its reabsorption after AVR, and pointed out the need to reconsider the timing of surgery in patients with concomitant CAD [46].

## Non-hemodynamic factors

Many factors are recognized as influencing the sequence of biological events that lead to the development of LV hypertrophy. Hemodynamic load is the fundamental stimulus, but genotype, gender and other not fully determined genetic and environmental factors regulate the growth of LV mass by means of proto-oncogenes, growth factors, neurohormones and cytokines [47]. The degree of the resulting structural changes, which may be compensatory or inappropriate but are probably always pathological [10], may influence the post-AVR remodeling process. It is in fact known that the regression potential of a hypertrophied LV is only partially influenced by improved hemodynamics, and some authors have therefore investigated the role of preoperative ultrastructural myocardial abnormalities. Lund *et al*. have conducted many studies in this field and, in 1998, published an interesting paper in which the findings from transmural biopsies taken during AVR were related to instrumental results after 18 months and to medium/long-term clinical outcomes [42]. Generally, a high nucleus volume, muscle cell mass index and fibrous tissue mass index were related to advanced disease characterized by impaired LV systolic and diastolic function, whereas the aortic valve gradient and wall stress did not correlate with any of the histological variables. Eighteen months after AVR, the LV mass index had decreased significantly, but the relative mass reduction was unrelated to the postoperative peak Doppler gradient, the diameter of the orifice of the prosthesis or the type of valve (which were not evaluated using the indexed EOA method). On the contrary, favorable remodeling was inversely related to muscle cell diameter, nucleus volume, percent fibrosis, the muscle cell mass index and the fibrous tissue mass index observed at the time of AVR, with the first two being the foremost determinants. Moreover, only 17% of the patients had a normal ventricular mass, although significant hypertrophy regression did take place. The extent of this regression after the removal of the hemodynamic trigger therefore seems to be predetermined by the presence of presumably irreversible myocyte abnormalities despite successful AVR [42]. In particular survival was inversely related to myocardial nucleus size (Fug.2). Prolonging the observation period and charting the time course of the LV mass index confirmed the pattern of regression, which was highly significant during the first 1.5 years, after which there was no further change up to 10 years. A lower LV mass index after 1.5 years was therefore a better predictor of long-term survival [17]. The potential for regression is the crucial point when evaluating the correct timing of surgery in patients with AS. The prognostic index developed by Lund *et al*. can estimate this potential because of its significant correlation with the LV mass index and long-term survival [17].

We have also searched for preoperative factors affecting absolute LV mass regression and, after an intermediate follow-up, we found that a higher preoperative LV mass was an independent non-hemodynamic predictor of greater regression (p < 0.0001), a finding that was also confirmed in patients with PPM [18,19]. This may explain why LV mass significantly regresses even in patients receiving a small prosthesis, although it does not necessarily mean that the regression is optimal or complete. Analysis of the data coming from a longer follow-up study currently coming to an end at our institution should further elucidate our preliminary findings concerning the role of preoperative non-hemodynamic factors. Hanayama *et al*. have recently found that, in addition to male gender, the extent of preoperative hypertrophy was the most important predictor of incomplete mass regression in a large study group and, like Gaudino *et al*. and Lund *et al*. previously [15,17], suggested that earlier surgical intervention may reduce the extent of postoperative residual hypertrophy and thus improve the outcome [17].

## Conclusion

Over the last 50 years, aortic valve surgery has made enormous strides towards the durable and physiological performance of aortic valve prostheses, thus allowing the restoration of normal LV structure and function. Interactions between surgeons and physicians have not only led to improvements in operative techniques and results, but have also facilitated a better knowledge of LV pathophysiology. We now understand much more about the nature of myocardial adaptation to pressure overload and myocardial responses to AVR, but some limitations need to be considered. The literature pertaining to the effects and efficacy of surgery largely consists of heterogeneous studies of relatively small populations, and differences in terms of patient selection, evaluated outcomes, operative interventions, and the timing of postoperative follow-up examinations limit their general applicability. Even the apparent simplicity of evaluating LV hypertrophy by means of echocardiography hides some intrinsic and largely unrecognized critical steps that may sometimes limit its clinical validity [48]. One positive note is that some of the discrepancies (such as the prevalence of PPM) are only apparent because an in-depth analysis may reveal that certain types of prosthesis are no longer implanted: for example, some studies included patients receiving the bulky and no longer available Starr-Edwards ball-caged valve [17], whereas many surgeons are now opting for supra-annular prostheses that allow improved hemodynamics even in small aortic annuli, and so better results can be expected in the future [49]. Other surgical options for minimizing PPM are also available, such as the replacement of the entire aortic root or the aortic root enlargement procedure described by Castro *et al*. [50], which bears the same operative risk as standard AVR and minimizes the incidence of PPM. In our institution, we have concentrated on the question of subvalvular obstruction because it has been reported that fixed or dynamic obstruction of the LV outflow tract after AVR is responsible for residual symptoms and incomplete hypertrophy regression, and found that a strategy including myectomy-myotomy before prosthetic valve implantation positively influences LV mass regression and favorable LV remodeling (Additional File 1) [51].

Other often-overlooked factors affecting the postoperative course are now emerging, such as ethnicity or arterial hypertension [15,48,52,53]. In particular, high blood pressure after AVR is gaining increasing attention as a strong and independent determinant of slower and incomplete mass regression. Research is also going forward at cellular level, and we early demonstrated that myocyte hyperplasia significantly contributes to LV hypertrophy: the increased cardiac mass associated with human AS is due to a combination of myocyte hypertrophy and hyperplasia, and intense new myocyte formation takes place as a result of the differentiation of stem-like cells committed to the myocyte lineage in response to an increased workload [54]. These findings, together with the identification of new environmental or genetic factors, will lead to new interpretations of the maladaptative mechanism of LV hypertrophy and the process of mass regression at cellular level. Meanwhile randomized comparison of stentless versus stented valves failed to affirm the superiority of stentless prosthesis. Actually, despite significant differences in indexed EOA and peak flow velocity in favor of the stentless valve, there were similar reduction in LV mass at 6 months with both stented and stentless valves, which persisted at 12 months [55].

Post-AVR residual hypertrophy is a complex and important phenomenon and, although its incidence is decreasing, it still represents a vexing problem that has an impact on morbidity and, probably, mortality. There is no doubt that meticulous blood pressure control and an optimized drug regimen are fundamental, but the curative potential of surgery in AS may only be fully revealed by adopting a different timing for AVR. Earlier surgery can also be considered for asymptomatic patients with moderate AS and a low expected operative mortality who show a marked hypertrophic myocardial response to the increased afterload. No clinical trial has yet compared an early surgical strategy based on the degree of myocardial hypertrophic remodeling with the traditional timing mainly based on symptoms and valve parameters, although the former makes sense as it has been shown that preserved or supranormal LV chamber performance can mask myocardial tissue dysfunction and surgery could hypothetically prevent it. However, surgery has already improved the prognosis of patients with AS, and many intraoperative options are now available to tailor the right operation for each patient. In our experience, every effort should be made if a small projected indexed EOA is expected to choose a prosthesis with the best projected indexed EOA and implant it adequately by adding other procedures, such as myotomy-myectomy, that allow favorable remodeling and the long-term benefits of LV mass regression.

## Competing interests

The author(s) declare that they have no competing interests.

## Contributions

All authors contributed to the paper and meet the criteria for authorship. All authors read and approved the final manuscript.

**Figure 2:**
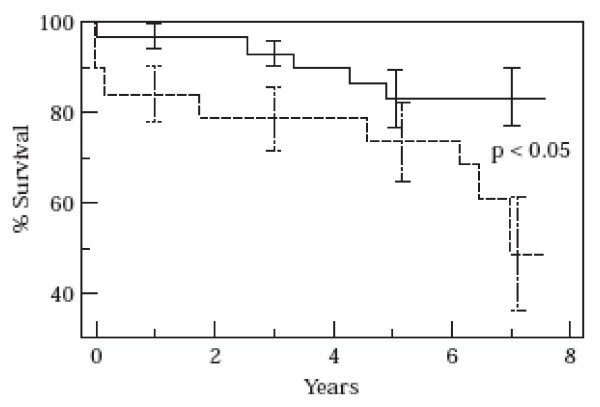
**Influence of LV histological findings on outcome after AVR**. Cumulative survival after the operation in relation to muscle cell nucleus volume. Five- and 7-year survivals were 83 ± 7% and 83 ± 7%, respectively, for a nucleus volume of ≤ 820 μm^3 ^( ____ ), and 74 ± 10% and 49 ± 14%; respectively, for a nucleus volume of >820 μm^3 ^( ------ ). *(Lund O, et al. Myocardial structure as a determinant of pre- and postoperative ventricular function and long-term prognosis after valve replacement for aortic stenosis. Eur Heart J 1998,19:1099–1108, by permission of Oxford University Press) *[42].

**Table 1:** Normal reference values of EOA for the prosthetic aortic valves. EOA is expressed as mean values available in the literature

	No. of Patients,* %	Prosthetic Valve Size, mm					
		
		19	21	23	25	27	29
							
Stented bioprosthetic valves							
Medtronic Intact	129 (10.2)	0.85	1.02	1.27	1.40	1.66	2.04
Medtronic Mosaic	390 (30.8)	1.20	1.22	1.38	1.65	1.80	2.00
Hancock II	53 (4.2)	...	1.18	1.33	1.46	1.55	1.60
Carpentier-Edwards Perimount	59 (4.7)	1.10	1.30	1.50	1.80	1.80	...
St. Jude Medical X-cell	21 (1.7)	...	...	...	...	...	...
Stentless bioprosthetic valves							
Medtronic freestyle	368 (29.1)	1.15	1.35	1.48	2.00	2.32	...
St Jude Medical Toronto SPV	60 (4.7)	...	1.30	1.50	1.70	2.00	2.50
Mechanical valves							
St Jude Medical Standard	151 (11.9)	1.04	1.38	1.52	2.08	2.65	3.23
St Jude Medical Regent	13 (1.0)	1.50	2.00	2.40	2.50	3.60	4.80
MCRI On-X	18 (1.4)	1.50	1.70	2.00	2.40	3.20	3.20
Carbomedics	3 (0.2)	1.00	1.54	1.63	1.98	2.41	2.63
Björk Shiley CC	1 (0.1)	...	...	...	...	...	...

* No. of patients with the prosthesis in the cited study [27]. *(Modified from Blais C, et al. Impact of valve prosthesis-patient mismatch on short-term mortality after aortic valve replacement. Circulation 2003:108:983–988, by permission of LWW) *[27].

## Supplementary Material

Additional File 1 Myectomy-myotomy before prosthetic valve implantation. (The sequence is extrapolated from an aortic root replacement – Bentall operation and the excision is performed in the proximal interventricular septum under the commissure between the ablated left and right aortic cusp). Click here for file
